# Effects of ingestion of a commercially available thermogenic dietary supplement on resting energy expenditure, mood state and cardiovascular measures

**DOI:** 10.1186/1550-2783-10-25

**Published:** 2013-04-30

**Authors:** Jordan Outlaw, Colin Wilborn, Abbie Smith, Stacie Urbina, Sara Hayward, Cliffa Foster, Shawn Wells, Rob Wildman, Lem Taylor

**Affiliations:** 1 Human Performance Lab, Department of Exercise and Sport Science, University of Mary Hardin-Baylor, Human Performance Lab, Belton, TX, 76513, USA; 2 University of North Carolina at Chapel Hill, Chapel Hill, NC, 27514, USA; 3 Dymatize Nutrition & Sport Performance Institute, 13537 N. Stemmons Fwy, Dallas, TX, 75234, USA

**Keywords:** Caffeine, Thermogenic, Resting energy expenditure

## Abstract

**Background:**

Increasing metabolism is a primary focus of many commercially available dietary supplements marketed to support weight management. Caffeine (e.g. anhydrous and herbal) and green tea are key ingredients in such products, augmenting resting energy expenditure (REE) and improving reported mood states (alertness, fatigue, focus, etc.). The purpose of this study was to evaluate the effects of a thermogenic dietary supplement (DBX) on REE, respiratory exchange ratio (RER), reported measures of alertness, focus, energy, concentration, fatigue, and hunger, as well as the general safety of the product based on electrocardiogram (ECG) and hemodynamic responses in habitual caffeine consumers.

**Methods:**

Six male and six female subjects (mean ± SD; 22.50 ± 3.22 years; 76.94 ± 14.78 kg; 22.7 ± 9.5% body fat), physically active (≥12 months), and moderate habitual caffeine consumers (<200 mg/day) received either two capsules of DBX containing 340 mg of total caffeine plus green tea extract, yerba mate extract, carnitine tartrate and other active ingredients or a placebo (PLC) in a double-blinded, crossover design. Heart rate (HR), blood pressure (BP), REE, RER and perceived mood states were measured at baseline and then hourly for four hours after ingesting either treatment.

**Results:**

Resting energy expenditure was significantly increased at all four time points and significant increases were determined for perceived alertness (p = 0.026) and focus (p = 0.05) at hour 1 and for energy at 1 and 2 hours after treatment for the DBX group (p = 0.008 and p = 0.017, respectively). Additionally, perceived fatigue was decreased at the hour 1 assessment (p = 0.010). No significant differences were seen between DBX and placebo for hunger, anxiety, HR, BP, ECG patterns or RER.

**Conclusions:**

The results of this investigation support that the proprietary blend of this thermogenic aid is capable of increasing REE for four hours post-ingestion while supporting increased focus, alertness, and energy as well as decreasing fatigue without promoting anxiety or causing significant changes in HR, BP, or ECG measurements in habitual caffeine consumers.

## Background

Augmentations in overall metabolism and “fat burning” are two physiological expectations of consumers when purchasing a thermogenic dietary supplement. One of the primary reasons for taking a thermogenic aid is to support weight loss and body leaning [1]. Many of these products found on the market, and available to the general public, contain synthetic caffeine and herbal sources (e.g. guarana, yerbe mate), green tea extract, and other purported metabolic-supporting ingredients such as carnitine and capsaicin (red pepper extract). The most researched ingredient, caffeine, has been reported to have several potential ergogenic benefits including increased energy expenditure and weight loss, decreased body fat, as well as increased performance and a potential glycogen-sparing effect [2,3]. Green tea extract with a standardized level of catechins in combination with caffeine has been shown to significantly increase daily energy expenditure and fat oxidation over that of caffeine alone [4]. Rudelle and associates [5] investigated the effects of a thermogenic drink containing green tea catechins, as well as caffeine, on energy expenditure in lean individuals. The beverage increased resting energy expenditure (REE) by 4.6% and the authors suggested that this type of beverage could be beneficial for weight loss and management. The increase in energy expenditure reported by multiple researchers [6-9] positions caffeine and green tea-containing supplements as a beneficial tool to offset the reduction in energy expenditure associated with weight loss [10-12]. In addition to affecting metabolism and favoring fat as a fuel source, many studies have shown that caffeine has an impact on alertness, fatigue, and other mood states [13-15]. After ingesting 120 mg of caffeine supplementation, greater alertness was reported for up to three hours by Mitchell and colleagues [13] and 40 mg of caffeine combined with 97 mg of L-theanine, the key caffeine analog in tea, showed improvements in perceived alertness and tiredness 20 and 70 minutes after ingestion in an investigation led by Giesbrecht and associates [14]. Caffeine levels of 250 mg and 500 mg also decreased reported tiredness and increased self-reported alertness when given to nine healthy subjects [15]. One important consideration in caffeine consumption studies is the control of habitual intake as individuals can become acclimated to caffeine, thus influencing their physiological responses to a specific dose.

Seeing these potential benefits for their consumers, supplement companies have created their own proprietary blends for weight management and body leaning supplements, as well as ergogenic aids containing caffeine. Many of these products claim to increase metabolism and “fat burning” either independently, or in conjunction with the caffeine contained in the supplement. Because of the popularity of weight management supplements, researchers have investigated different thermogenic products to determine their effectiveness. For instance, Hoffman and colleagues [16] determined that a commercially available product containing multiple trademarked ingredient mixtures demonstrated a trend for increased fat oxidation while also increasing heart rate (HR), systolic blood pressure (SBP) and reported levels of tension and confusion among the supplement group. Another study performed in 2009 [17] revealed that capsaicin, an active ingredient in the DBX proprietary blend, statistically increased energy expenditure and diastolic blood pressure (DBP) after ingestion but had no influence on fat utilization. A third investigation [18] revealed a significant effect for REE, as well as RER and SBP over three hours when regular coffee drinkers consumed a commercial product containing roughly 400 mg total caffeine.

Based on these past studies, many thermogenic supplements are successful at increasing energy expenditure, but varying doses and combinations of ingredients may cause different cardiovascular and mood state side effects. Further product-specific research on thermogenic aids is needed to determine levels of effectiveness and safety for consumers. The purpose of this study was to evaluate the effects of a commercially available thermogenic dietary supplement on energy expenditure, reported measures of alertness, focus, energy, concentration, fatigue, and hunger, as well as the general tolerance and safety of the supplement based on ECG and hemodynamic responses when taken by healthy, active, young adults.

## Methods

### Participants

Six males and six females (mean ± SD; age: 22.50 ± 3.22 years; weight: 76.94 ± 14.78 kg; body fat: 22.7 ± 9.5%) volunteered for the study conducted in the Human Performance Lab (HPL) at the University of Mary Hardin-Baylor in Belton, Texas. Participants were required to be apparently healthy, physically active (regularly participating in exercise for the previous 12 months), moderate caffeine users (<200 mg/day), and were excluded from the study if they had any known metabolic disorders, were sensitive to caffeine, had a history of pulmonary disease, hypertension, liver or kidney disease, musculoskeletal or neuromuscular disease, neurological disease, autoimmune disease, or any cancers, peptic ulcers, or anemia. Taking certain medications, including those for heart, pulmonary, thyroid, anti-hyperlipidemic, hypoglycemic, anti-hypertensive, endocrinologic, psychotropic, neuromuscular, neurological, or androgenic conditions, as well as a family history of heart problems, high blood pressure, and/or stroke, and being pregnant or breastfeeding were also factors for exclusion. Trained lab assistants screened and examined participants as well as obtained a complete medical history to determine if each participant met the qualification standards. Participants reported the number of caffeinated beverages (coffee, tea, soft drink, energy drink, etc.), caffeine containing medications (NoDoz, Vivarin, etc.) and caffeine containing foods (candy, chocolate ice cream, etc.) as well as the serving size (8 oz., 5 oz., etc.) of each reported caffeinated product they consumed per week on average. Average caffeine consumption was determined to be 176.59 ± 86.63 mg/day. Volunteers were required to report any previous or current use of nutritional supplements, prescription and non-prescription medications. Participants were instructed to not change their nutritional supplement/medication intake over the course of the study and to report any changes to lab personnel.

### Instruments

#### Anthropometric measures

Body composition was determined with the use of the Discovery QDR Dual-Energy X-ray Absorptiometry (DEXA) machine (Hologic, Inc., Bedford, MA). Participants were positioned in a supine position on the DEXA machine, in either workout shorts or hospital gown, and were asked to remain still during the entirety of the six minute scan. Percent body fat (%Fat), fat free mass (FFM; grams), and fat mass (FM; grams) were collected from the DEXA report. Height was obtained from the SECA 242 measuring instrument (242, SECA, Hanover, MD) and recorded in both centimeters and inches. The TANITA Body Composition Analyzer (Model TBF-310, TANITA, Arlington Heights, IL) was utilized to measure weight in both kilograms and pounds.

#### Resting energy expenditure

REE was measured using a TrueOne® 2400 metabolic measurement system (ParvoMedics, Sandy, UT). The metabolic cart was calibrated daily by trained laboratory assistants according to manufacturer guidelines. During testing, participants rested in a supine position with a blanket in a quiet, semi-dark room. A clear hood was placed over the participant’s head and upper torso area. REE and respiratory exchange ratio (RER) data were collected from the last 20 minutes of the 25 minute test. For each breath, mean oxygen uptake (VO_2_) and carbon dioxide output (VCO_2_) were measured and then averaged over 15 second intervals. Flow rate was monitored by lab assistants during the course of the test and maintained at a rate of 1–1.2 L/min of expired carbon dioxide. The test-retest correlations (r) of this metabolic cart range from 0.814-0.956 [19].

#### Mood state questionnaire

A 5-point Likert scale questionnaire was used to measure perceived alertness, focus, energy, fatigue, concentration, and hunger. The participant placed a check mark in the specific box that correlated with their perceived mood level for all six categories. The numbers ranged from one (not feeling that particular mood) to five (highest level of mood).

#### Hemodynamic assessments

Electrocardiogram (ECG) leads were placed in standard clinical fashion to reveal 12 leads (I-III, V_1_-V_6_, aVR, aVL, aVF) throughout the testing session. Cardiac rhythm was monitored through a Quinton Eclipse Premier Electrocardiograph (Cardiac Science Corporation, Bothell, WA). Every five minutes, data were printed from the 12-lead ECG machine and RR interval, RP interval, QRS duration, and QT interval were recorded. If any abnormal readings/tracing were discovered, a note was added to the patient’s file. Heart rate, recorded as beats per minute and SBP and DBP, recorded as mmHg, were measured at baseline and hourly for four hours after consuming either treatment.

#### Diet log

Participants were instructed to maintain a diet log for four days prior to the first testing session, testing day one, as well as days between testing sessions. Lab personnel instructed participants to report foods eaten at breakfast, lunch, and dinner, as well as snacks. They were also instructed to record the method of preparation for each food and the quantity eaten (servings, cups, tablespoons, etc.). Trained HPL assistants input the data from the diet logs into the Food Processor Nutrition and Fitness Software (ESHA Research, Salem, OR) computer program, which was used to determine caloric intake based on reported information from participants.

#### Supplement

Our active supplement Dyma-Burn® Xtreme (Dymatize Enterprises, LLC, Dallas, TX) contains multiple ingredients combined to provide metabolic support including caffeine anhydrous, guarana, yerba mate green tea extract, L-carnitine L-tartrate (200 mg), pathothenic acid (17 mg), chromium picolinate (100 mcg) and proprietary blends containins , AssuriTea™ Green Tea Extract (Kemin Nutritionals, Iowa City, IA), *Salvia sclarea*, raspberry ketones and *Capsicum Annum* extract, plus l-tyrosine, salix alba (white willow), zingiber officinale (ginger), focus vesiculosus (bladderwrack), panax ginseng, and Bioperine® (black pepper extract). The total caffeine and catechin content of the supplement was 340 mg and 60 mg respectively.

#### Procedures

Participants completed medical and exercise history surveys as well as signed an Informed Consent before beginning the study. Typical caffeine intake, over the counter drug usage, perceived fatigue, and appetite were reported along with daily caffeine consumption. All participants and paperwork were examined by qualified laboratory personnel. On the first day of the study, participants reported to the HPL at 8:00 am in a 12-hour fasted state. All testing sessions were held in the morning hours to reduce changes in REE due to performance of daily activities and stresses. This study was conducted in a double-blind, crossover manner with participants consuming either 2 capsules of a placebo (PLC) or 2 capsules of the active supplement (DBX). Before the initial treatment, DEXA was performed to assess body composition. Meanwhile, before either treatment, ECG electrodes were then positioned by HPL assistants and a baseline ECG was recorded. A 12 lead ECG printout was collected every five minutes throughout the testing period. A baseline metabolic test was conducted prior to supplementation and REE and RER data were recorded. After the initial REE session, each subject then consumed the randomly assigned treatment. Post supplementation, REE and RER data was collected from the last 20 minutes of the metabolic test at 60, 120, 180, and 240 minutes. At the end of testing day one, participants left the HPL and returned three days later to complete another testing session identical to the first with the exception of consuming whichever treatment was not consumed on test day one. A timeline for the testing day can be seen in Table 1.

**Table 1:** Testing day timeline

**Testing day timeline**							
**DEXA**	×						
**REE (ending time)**			×	×	×	×	×
**ECG Begins**		×					
**Supplementation**			×				
**BP/HR**			×	×	×	×	×
**Mood State Ques.**			×	×	×	×	×
	**−45 min**	**−30 min**	**0 min**	**60 min**	**120 min**	**180 min**	**240 min**

REE testing began 25 minutes before the end of each hour and lasted for 25 minutes.

HR and BP were recorded at the end of each hour and participants completed a mood state questionnaire at this time point as well. ECG electrodes were placed before the baseline REE and data were printed every five minutes throughout the entirety of the testing session.

### Data analysis

All data was analyzed in SPSS using a mixed-factorial ANOVA [treatment (DBX vs PLC) x time (HR1 vs HR2 vs HR3 vs HR4)]. When a significant interaction was found, a lower order ANOVA with Bonferroni post-hoc corrections was used. A *Kruskal-Wallis* one-way analysis of variance was also used for all survey data. Significance was set at *p* ≤ 0.05.

## Results

### REE and RER

**Figure 1:**
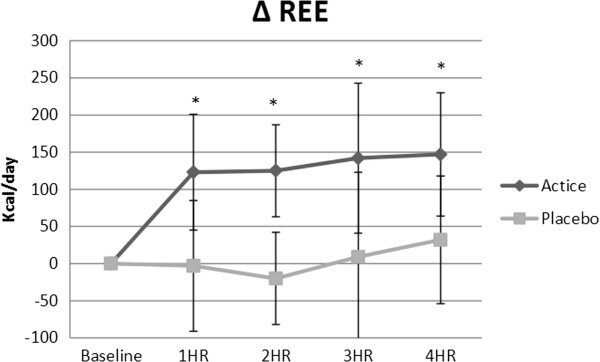
**Resting energy expenditure changes.** REE increased across all time points for DBX (active) ranging from a 123.4 to 147.3 kcal/day increase above baseline values. Changes were statistically different between groups at all time points post-supplementation. * indicates statistically significant changes (*p* ≤ 0.05).

A significant group x time interaction for change in resting energy expenditure (*p* = 0.001) was determined. From baseline to hour 4, REE increased by 147.33 ± 83.52 for DBX and 32.17 ± 86.72 kcal/day for PLC (*p* = 0.003). Changes in kcal/day for all time points can be seen in Figure 1. A significant main effect for time was also reported (p = 0.001). Changes in REE from baseline for each time point are as follows: hour 1 (DBX: 123.4 ± 78.2 kcal/day vs. PLC: -3.1 ± 88.4 kcal/day), hour 2 (DBX: 125.5 ± 62.2 kcal/day vs. PLC: -20.3 ± 72.6 kcal/day), hour 3 (DBX: 142.4 ± 101.16 kcal/day vs. PLC: 9 ± 114.77 kcal/day), and hour 4 (DBX: 147.3 ± 83.5 kcal/day vs. PLC: 32.1 ± 86.7 kcal/day). Changes were significant (p < .05) between groups at all time points for REE. There were no significant time or interaction effects for RER at any time point.

### Hemodynamic and ECG

**Figure 2:**
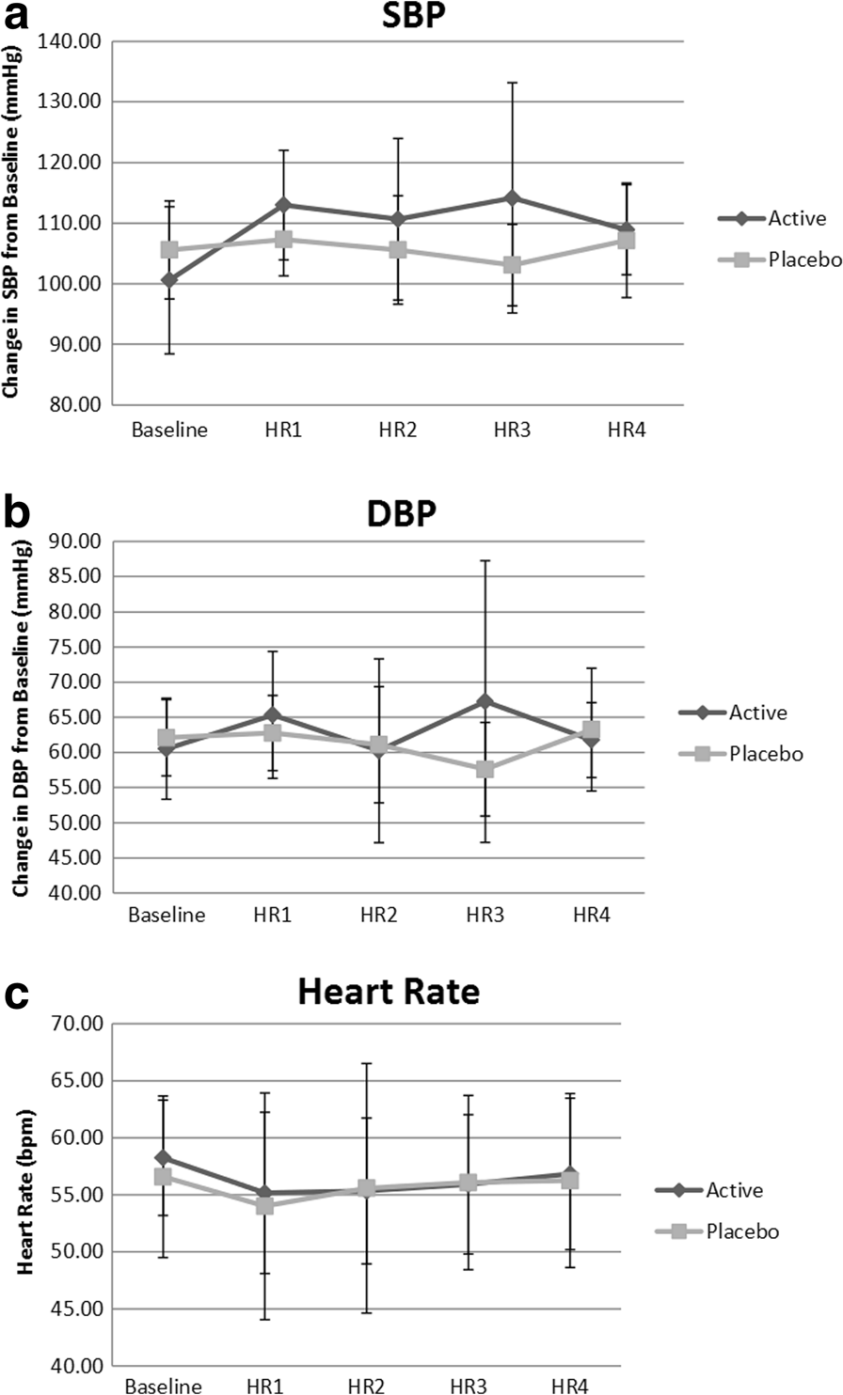
**Hemodynamic measurement changes. a**: Systolic Blood Pressure did not significantly differ from baseline values at HR1, 2, 3 or 4 for the active supplement group. **b**: Diastolic blood pressure did not significantly differ from baseline values at HR1, 2, 3 or 4 for the active supplement group. **c**: Heart rate, represented as beats per minute, was not significantly changed at any time point compared to baseline measurements for the supplement group.

There were no significant (*p* > 0.05) group x time interactions and no main effects for time for SBP, DBP, or HR (Figure 2). There was no significant main effect for group (*p* > 0.05). At hour 1, SBP increased by 12.4 ± 11.8 mmHG and 1.75 ± 10.4 mmHG for DBX and PLC, respectively from baseline values. From baseline to hour 2, SBP increased by 10.0 ± 14.0 mmHg (DBX) versus 0.0 ± 7.9 mmHg (PLC). Hour 3 SBP deviated from baseline by 13.5 ± 22.4 mmHg for DBX and −2.5 ± 8.1 mmHg for PLC. Hour 4 SBP increased above the baseline mean by 8.3 ± 10.5 mmHg (DBX) and 1.5 ± 10.6 mmHg (PLC). DBP changes from baseline to hour 1 were 4.8 ± 7.4 mmHg (DBX) versus 0.6 ± 7.9 mmHg (PLC). At hour 2, DBP changed from baseline by −0.25 ± 13.2 (DBX) and −1.0 ± 7.2 mmHg (PLC). Hour 3 values for DBP from baseline for DBX were 6.7 ± 20.9 mmHg and for PLC were −4.5 ± 10.1 mmHg. The comparison against DBP baseline measurement for the DBX group at hour 3 was 1.25 ± 6.8 mmHg and 1.1 ± 11.0 mmHg for the PLC group. DBX versus PLC comparison to baseline in HR are as follows: hour 1 (−3.0 ± 6.2 vs. -2.5 ± 5.5 bpm), hour 2 (−2.9 ± 6.5 vs. -1.0 ± 10.0 bpm), hour 3 (−2.3 ± 5.6 vs. -0.5 ± 8.7 bpm), and hour 4 (−1.4 ± 6.8 vs. -0.3 ± 7.4 bpm). (Data can be seen in Table 2 for SBP, DBP, and HR.) In addition to SBP, DBP, and HR, no significant differences in group or time were observed for the ECG intervals (RR, PR, and QT) or QRS duration. There were no observed changes in ECG rate and rhythm patterns.

**Table 2:** Hemodynamic Measures

**SBP, DBP, and HR Measurements Baseline to HR4**						
	**SBP mean ± SD (mmHg)**		**DBP mean ± SD (mmHg)**		**HR mean ± SD (bpm)**	
	**DBX**	**PLC**	**DBX**	**PLC**	**DBX**	**PLC**
**Baseline**	100.58 ± 12.12	105.58 ± 8.08	60.50 ± 7.20	62.08 ± 5.42	58.25 ± 5.07	56.58 ± 7.10
**HR1**	113.0 ± 9.04	107.33 ± 6.04	65.33 ± 9.03	62.75 ± 5.36	55.17 ± 7.09	54.00 ± 9.94
**HR2**	110.67 ± 13.36	105.58 ± 8.96	60.25 ± 13.06	61.08 ± 8.28	55.33 ± 6.41	55.58 ± 10.94
**HR3**	114.17 ± 19.00	103.08 ± 6.75	67.25 ± 20.01	57.58 ± 6.67	55.92 ± 6.11	56.08 ± 7.66
**HR4**	108.92 ± 7.44	107.17 ± 9.48	61.75 ± 5.33	63.25 ± 8.75	56.83 ± 6.64	56.25 ± 7.64

SBP, DBP, and HR were recorded at baseline, HR1, HR2, HR3, and HR4.

Measurements for SBP and DBP are reported as mean ± SD and recorded in units of mmHg. Changes in SBP and DBP were not significant at any time point for either group. Heart rate measurements were reported as mean ± SD and recorded in beats per minute. Changes in HR were not significant at any time point for either group.

### Subjective measures of mood state

**Figure 3:**
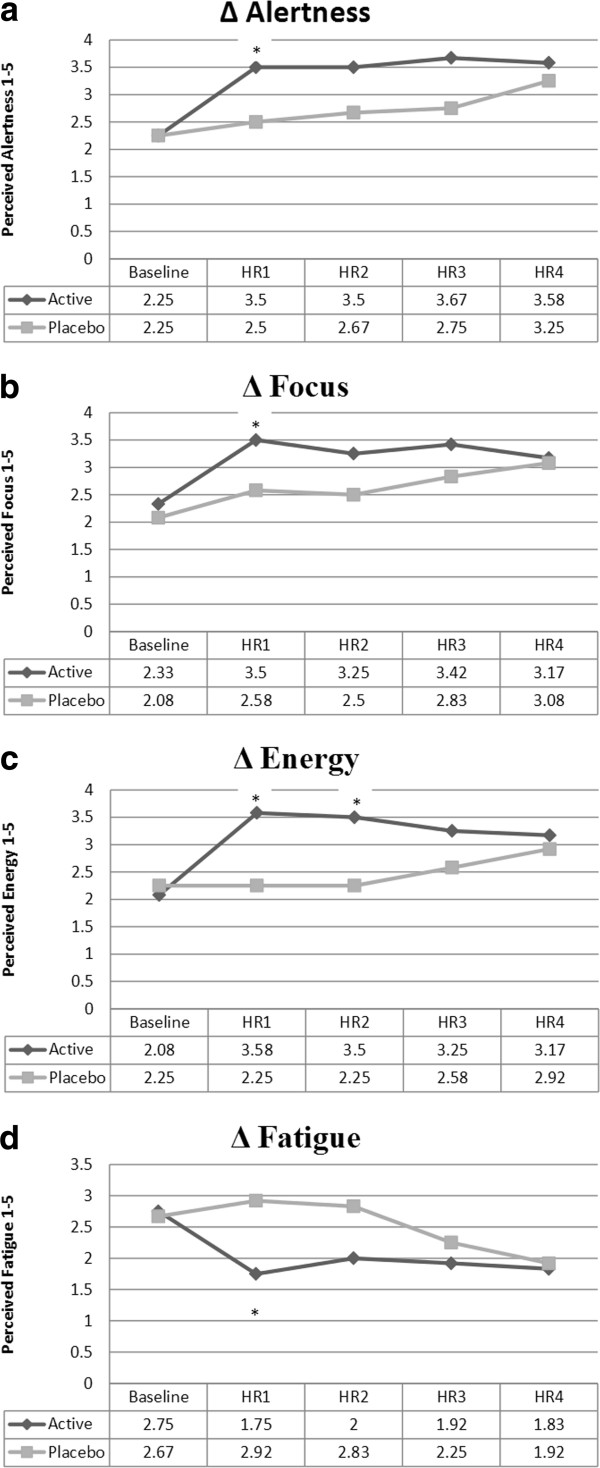
**Changes in reported mood states. a**: Alertness was reported on a 5-point Likert scale and rated one through five, five being the highest. Changes in alertness for the active supplement group were significant at HR1 only. * indicates statistically significant changes (*p* ≤ 0.05). **b**: Focus was reported on a 5-point Likert scale and rated one through five, five being the highest. A significant increase in focus was seen at HR1 for DBX. * indicates statistically significant changes (*p* ≤ 0.05). **c**: Energy was reported on a 5-point Likert scale and rated one through five, five being the highest. Changes in perceived energy were significant at both HR1 and HR2 for the supplement group. * indicates statistically significant changes (*p* ≤ 0.05). **d**: Fatigue was reported on a 5-point Likert scale and rated one through five, five being the highest. Decreases in fatigue were significant for the supplement group at HR1. * indicates statistically significant changes (*p* ≤ 0.05).

Significant within group increases (*p* < 0.05) were observed for both alertness (*p* = 0.026) and focus (*p* = 0.05) at hour 1 and energy at hour 1 (*p* = 0.008) and 2 (*p* = 0.017) for DBX. Within group decreases in fatigue were observed for fatigue for the DBX group at the hour 1 time point, and no significant within group changes occurred for either hunger or concentration (*p* > 0.05). Mood state data can be seen in Figure 3.

## Discussion

The results from this study indicate that this particular thermogenic aid is capable of significantly increasing REE (+8%) for at least four hours post-ingestion in moderate-level habitual caffeine consumers. It is reasonable to contribute the increase in REE to the 340 mg proprietary blend of caffeine anhydrous, guarana, yerba mate, and green tea extract found in the commercially available DBX. Caffeine is a known stimulant and increases energy expenditure and weight loss. In combination with catechins, caffeine has been proven to decrease body fat percentage and waist circumference in overweight individuals [20]. Increased fat utilization as a fuel source is another benefit often associated with caffeine ingestion and supplementation. A 2011 meta-analysis [21] concluded that while caffeine ingestion increases energy expenditure, it appears to be unable to increase fat oxidation unless paired with catechins. Fat oxidation was significantly increased when 375 mg of catechin was paired with 150 mg of caffeine [22], 540 mg catechin with 300 mg of caffeine [5], and when 662.5 mg of catechin was consumed with 270 mg of caffeine [23]. The current study contradicts conclusions reported by Hursel and colleagues [13] as the Dyma-Burn® Xtreme supplement does contain a catechin-caffeine mixture but RER was not significantly changed over the four hour testing period (*p* > 0.05). This could possibly be explained by the lower level of catechin (50 mg) used in this particular product. More so, differences may be attributed to the use of both men and women with varying resting RER levels. The results of this investigation suggest that while DBX can promote a rapid and sustained increase in REE, the increase is not due to enhanced fat oxidation. Research from 2001 [24] supports the RER data from the current investigation as Graham concludes that caffeine’s role as a glycogen sparing aid is not fully supported by research.

The active supplement promoted increases in perceptions of alertness, focus, and energy, and also decreased fatigue without impacting perceived anxiety levels. These findings suggest that this product might have a favorable impact on the perceived quality of daily activities including exercise. Here again, caffeine is the most studied of the active ingredients and believed to be the main contributing factor to the positive changes in alertness, focus, energy, and fatigue. In a study by Zwyghuizen-Doorenbos and colleagues [25], a dosage of 250 mg of caffeine increased alertness in healthy young men. Those consuming the caffeine also performed better than those who received the placebo. With this in mind, this supplement may be beneficial for persons looking to burn more calories throughout the day and increase exercise performance. Furthermore, the experimental design of this study was based on an acute response and it is unclear at this time what the effects might have on chronic exercise and training adaptations when the product is taken for several weeks.

Of interest is the potential real world application of this study considering all of the participants were habitual caffeine consumers with a moderate daily intake of caffeine (<200 mg/day) and were still responsive to the active supplement treatment. This regular intake of moderate amounts of caffeine may explain much of the lack of observed hemodynamic and ECG effects in this investigation. Tolerance to caffeine can develop within four days of consuming 150 mg/day [26] and this built-up tolerance can negate or reduce the side effects often seen when a non-caffeine user ingests a caffeine-containing beverage/supplement including increases in SBP, DBP, and changes in HR [27]. In addition to a lack of negative physiological side effects, participants also did not report any negative mood states or other side-effects. When participants were given 280 mg of caffeine in the form of coffee, Smits and associates [28] observed an increase in BP and a decrease in HR, while there were no significant changes among the control group (decaffeinated coffee). These changes in HR and BP were assumed to be linked to the caffeine content of the regular coffee. Considering the supplement used in the present study contained 340 mg of total caffeine, habitual moderate caffeine usage seems to be the contributing factor to no significant changes in HR, BP, and ECG data, as well as the lack of reported side-effects.

## Conclusion

In conclusion, when taken by moderate caffeine users that are physically active and healthy, the proprietary blend of this particular thermogenic supplement can increase REE and mood states related to alertness, focus, and energy without causing unsafe acute hemodynamic side-effects or increasing perceived anxiety levels. Future research should evaluate the chronic combined effects of DBX with exercise.

## Abbreviations

REE: Resting energy expenditure; RER: Respiratory exchange ratio; ECG: Electrocardiogram; HR: Heart rate; BP: Blood pressure; SBP: Systolic blood pressure; DBP: Diastolic blood pressure; HPL: Human performance Lab; DEXA: Dual-Energy X-ray Absorptiometry; %Fat: Percent body fat; FFM: Fat-free mass; VO2: Mean oxygen uptake; VCO2: CO_2_ output.

## Competing interests

Shawn Wells and Rob Wildman are employees of Dymatize Inc. Dymatize Inc. was the study funder. Neither contributor was involved in data collection or analysis. Their involvement was limited to manuscript preparation.

## Authors’ contributions

JO was the primary author and prepared the manuscript. CW was the primary investigator and designed the study. CW, AS, SW, and RW assisted with manuscript preparation. SU, SH, and LT conducted all testing and statistical analysis. CF provided administrative oversight. All authors read and approved the final manuscript.
